# *RDL* mutations in Guangxi *Anopheles sinensis* populations along the China–Vietnam border: distribution frequency and evolutionary origin of A296S resistance allele

**DOI:** 10.1186/s12936-020-3098-y

**Published:** 2020-01-15

**Authors:** Nian Liu, Xiangyang Feng, Xinghui Qiu

**Affiliations:** 1 State Key Laboratory of Integrated Management of Pest Insects and Rodents, Institute of Zoology, Chinese Academy of Sciences, Beijing, 100101 China; 2 Institute of Physical Science and Information Technology, Anhui University, Hefei, 230039 China; 3 Guangxi Zhuang Autonomous Region Centre for Diseases Control and Prevention, Nanning, 530028 China

**Keywords:** *Anopheles sinensis*, Guangxi Zhuang Autonomous Region, China–Vietnam border, Insecticide resistance, RDL, Gamma-aminobutyric acid receptor

## Abstract

**Background:**

Malaria is a deadly vector-borne disease in tropical and subtropical regions. Although indigenous malaria has been eliminated in Guangxi of China, 473 confirmed cases were reported in the Northern region of neighbouring Vietnam in 2014. Considering that frequent population movement occurs across the China–Vietnam border and insecticide resistance is a major obstacle in disease vector control, there is a need to know the genotype and frequency of insecticide resistance alleles in *Anopheles sinensis* populations along the China–Vietnam border and to take action to prevent the possible migration of insecticide resistance alleles across the border.

**Methods:**

Two hundred and eight adults of *An. sinensis* collected from seven locations in Guangxi along the China–Vietnam border were used in the investigation of individual genotypes of the *AsRDL* gene, which encodes the RDL gamma-aminobutyric acid (GABA) receptor subunit in *An. sinensis*. PCR-RFLP (polymerase chain reaction-restriction fragment length polymorphism) analysis was deployed to genotype codon 345, while direct sequencing of PCR products was conducted to clarify the genotypes for codons 296 and 327 of the *AsRDL* gene. The genealogical relation of *AsRDL* haplotypes was analyzed using Network 5.0.

**Results:**

Three putative insecticide resistance related mutations (A296S, V327I and T345S) were detected in all the seven populations of *An. sinensis* in Guangxi along the China–Vietnam border. The resistance-conferring A296S mutation was found to be widely distributed and present at notably high frequencies (78.8% to 100%). Relatively lower frequencies of mutations V327I (26.9% to 53.2%) and T345S (0% to 28.8%) were observed. The V327I or T345S always occurred in the presence of A296S. Evolutionary analysis of 21 AsRDL haplotypes indicated multiple origins of the A296S and V327I mutations.

**Conclusion:**

The resistance A296S allele was present at high frequencies in the *An. sinensis* populations along the China–Vietnam border, indicating a risk of resistance to insecticides targeting RDL. The double mutations (A296S + V327I) may have evolved from alleles carrying the A296S mutation by scaffolding the additional mutation V327I, and A296S allele may have multiple evolutionary origins. These findings will help inform strategies for vector control and malaria prevention.

## Background

Malaria is one of the most serious vector-borne diseases. Despite being preventable and treatable, malaria continues to be a major threat to global public health. In 2017, there were an estimated 219 million cases of malaria and 435,000 deaths from malaria globally, with 5% of the cases occurring in the WHO South-East Asia Region [1]. Guangxi Zhuang Autonomous Region of China was once a malaria-endemic region [2]. After continued efforts for decades, the malaria burden has been substantially reduced in Guangxi [2, 3]. However, the risk of malaria re-emergence remains, due to the importation of malaria parasites in thousands of infected travellers from Africa, and Southeast Asia. In neighbouring Vietnam, the malaria incidence declined significantly between 1991 and 2014, but there were 14,941 confirmed cases in 2014, including 473 confirmed cases in the Northern region [4].

Insecticide-based vector control remains a key preventive strategy in the fight against malaria, credited with the significant reductions in malaria morbidity and mortality since 2000 due to the widespread implementation of insecticidal interventions [5]. However, the continued effectiveness of this strategy has been challenged by the increasing resistance of vectors to available insecticides. To minimize the risk of control failure caused by insecticide resistance, a better understanding of the status of and the genetic mechanisms underpinning resistance to commonly used insecticide is an urgent need.

In the previous study [6], the distribution and frequency of genetic mutations in two targets, acetylcholinesterase (AChE) and the voltage-gated sodium channel (VGSC), were investigated. The G119S mutation within AChE was present at high frequencies (0.61–0.85), but the *kdr* mutation was rare in the seven Guangxi *An. sinensis* populations along the China–Vietnam border, suggesting that pyrethroids remain suitable for use against *An. sinensis* [6]. However, to maintain their effectiveness, the application of pyrethroids should not be taken as the sole measure for vector control, thus insecticides with alternative modes of action should be considered.

The current study was a survey extended to another insecticidal target, gamma-aminobutyric acid (GABA) receptor subunit encoded by the *RDL* (Resistant to dieldrin) gene, which is the target of multiple types of insecticides, such as dieldrin (cyclodienes), fipronil (phenylpyrazoles), and fluralaner (isoxazolines) [7–9]. Efforts were given to detect naturally existing genetic mutations, map their distribution and frequency in seven *An. sinensis* populations along the China–Vietnam border, and track the origin of the resistance-related mutations. This work focused on amino acid substitutions at three positions (A296G/N/S, V327I and M345M/S) in RDL that are associated with insecticide resistance previously documented in multiple insect species [8–13]. The data obtained in this study are of significance for current and future malaria control programs given that the use of GABA targeting insecticides is on the increase.

## Methods

### Samples

*Anopheles sinensis* adults were caught in seven different sites from April 2015 to August 2017. The seven sample-collecting sites were located in different villages along the China–Vietnam border. Rice is the main crop planted in these villages. The commonly used insecticides for rice pest control in these areas are diamides (e.g. chlorantraniliprole), neonicotinoids (e.g. imidacloprid) and organophosphorates (e.g. acephate and dimethoate). Methods about sample collection and species identification were described by Yang et al. [6].

### *AsRDL* genotyping

A fragment of the *AsRDL* gene that includes codons 296 and 327 (named as RDL7) was amplified using primers (Primers ARE-7F and 7I-R, Table 1) and individual genomic DNA as template. The amplification reaction consisted of 25 μl of 2× Taq PCR Master Mix, 0.2 μM each primer, and 50–150 ng of DNA template in a total volume of 50 μl. The amplification was programmed as 95 °C for 5 min; 42 cycles of 95 °C for 30 s, 62 °C for 30 s, 72 °C for 70 s; and 72 °C for 10 min. PCR products were directly sequenced (TSINGKE Biotech, Beijing, China). For clone sequencing, three to ten clones were sequenced. The sequences from clone sequencing were cross-checked with those from direct PCR product sequencing to clarify the two haplotypes in each heterozygote.

**Table 1:** Brief information about PCR

Primer name	Sequence	Annealing temperature (°C)	Amplicon size (bp)
ARE-7F	AGTTTGTACGTTCGATGGGTTA	62	476
7I-R	GGCAACAGTAAGCTATGTCGA		
8F-1	ATAATCGCCCCGGATCACC	56	167
8R-1	CCTGCTGCTTCTTCTGTTCC		

**Fig. 1:**
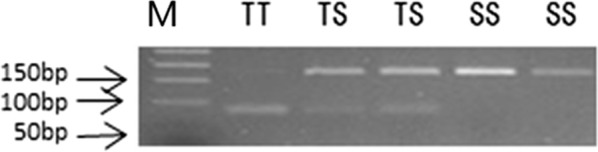
Diagnostic PCR-RFLP tests to detect the T345S mutation. The 167 bp amplicon is undigested by HpyCH4 III for homozygous TCG (mutant 345SS), and cut into two fragments of 83 bp and 84 bp for homozygous ACG (wild 345TT) that co-migrate in the agarose gel and cannot be distinguished. A combined pattern (two bands 167 bp + 83/84 bp) is displayed for heterozygotes

Primers 8F-1 and 8R-1 (Table 1) were used to amplify a fragment of 167 bp covering codon 345 of the *AsRDL* gene (named as RDL8). The reaction mixture (30 μl) included 2× Taq PCR MasterMix, 0.2 μM each primer and 30–100 ng of genomic DNA. The reaction procedure was set as 95 °C for 5 min; 38 cycles of 95 °C for 30 s, 56 °C for 30 s, 72 °C for 10 s; and 72 °C for 10 min. PCR products were digested with the restricted DNA digestion enzyme HpyCH4 III (New England Biolabs) (Fig. 1a) for 2 h in a total volume of 20 μl according to the manufacturer’s instruction. The digestion products were detected by 3% agarose gel electrophoresis. The genotype of each sample was identified based on the resulting gel profiles: one band of 167 bp for homozygous TCG (SS), one band of combined 83 bp and 84 bp for homozygous ACG (TT) (complete digestion of the 167 bp DNA by HpyCH4 III resulted in two fragments with a length of 83 bp and 84 bp respectively, which could not be distinguished on a routine agarose gel); two bands (167 bp + 83 bp) for T/ACG (S/T) heterozygote (Fig. 1b).

### Data analysis

Each sequencing result of RDL7 was manually checked and the terminal ambiguous regions were trimmed. All the confirmed sequences (360 bp) were aligned using Muscle 3.8 [14], and the polymorphic sites in the sequence were recorded. Hardy–Weinberg equilibrium (HWE) of genotypes in each population was tested by probability test using the online software GENEPOP v.4.2 [15]. The haplotypes of heterozygotes were clarified by clone sequencing. Haplotype network analysis was conducted using Network 5.0 [16].

## Results

### Nucleotide variations in the RDL7 region of the *AsRDL* gene

**Fig. 2:**
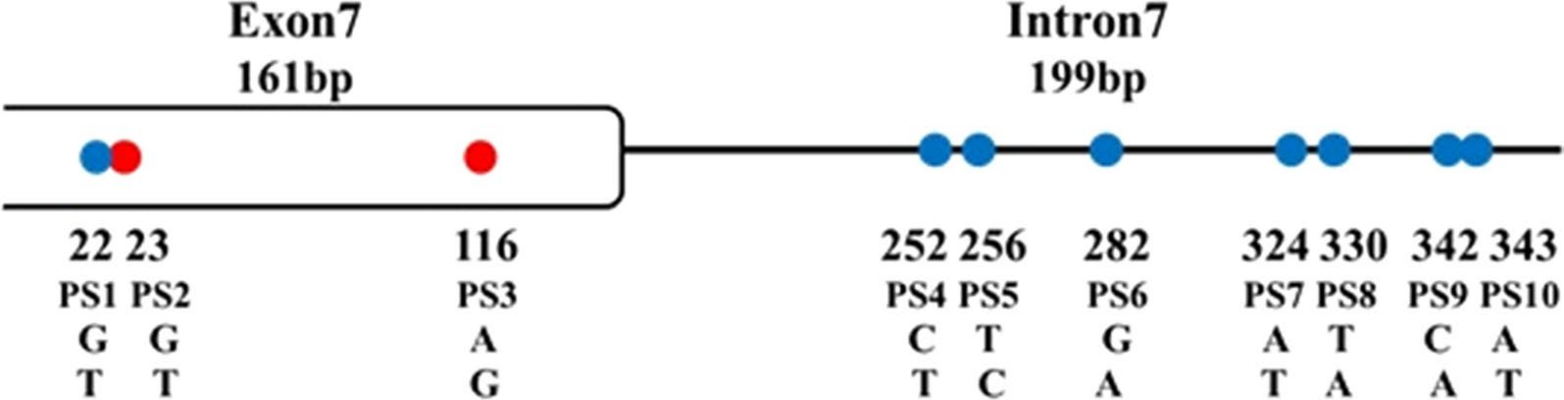
The polymorphic sites identified in the *AsRDL* gene in this study. Non-synonymous sites are indicated by red dots

Ten polymorphic sites (PS) were observed in RDL7 in a total length of 360 bp, three PSs in exon 7 and seven in intron 7 (Fig. 2). The 2nd and 3rd PSs represented synonymous mutations, causing amino acid substitutions at sites 296 (A296S) and 327 (V327I), respectively.

### Distribution and frequency of insecticide resistance-related mutations

**Fig. 3:**
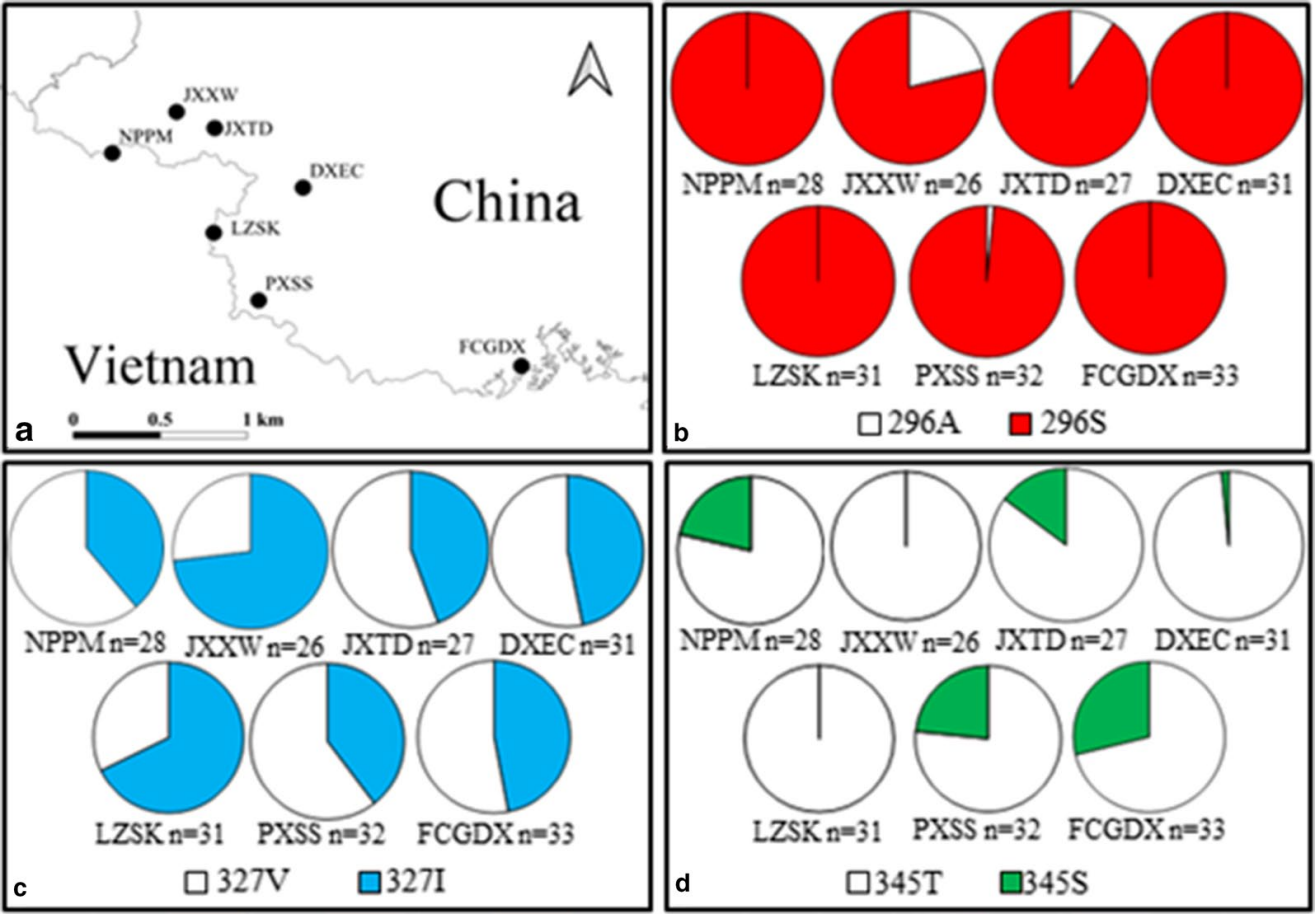
The sampling location (**a**), and distribution frequencies of AsRDL allele (**b**–**d**). *NPPM* Pingmeng, Napo County; *JXXW* Xinwei, Jingxi County; *JXTD* Tongde, Jingxi County; *DXEC* Encheng, Daxin County; *LZSK* Shuikou, Longzhou County, *PXSS* Shangshi, Pingxiang County; *FCGDX* Dongxing town, Fangchenggang City

The distribution and frequency of the three insecticide resistance-related mutations are shown in Table 2 and Fig. 3. Overall, the frequency of A296S was very high (79–100%) in the seven examined populations. Notably, this allele was fixed in four of the seven populations. By contrast, the frequency of the susceptible allele was rare: A296 was only detected in three populations (PXSS, JXTD, JXXW) at frequencies ranging from 1.5 to 21%.

**Table 2:** Frequency of *RDL* genotypes in seven *An. sinensis* populations along the China–Vietnam border

Population	n	Residue 296 genotype			HWE test	Residue 327 genotype			HWE test	Residue 345 genotype			HWE test
		AA	AS	SS		VV	VI	II		TT	TS	SS	
DXEC	31	0	0	31	–	6	17	8	0.4792	30	1	0	–
FCGDX	33	0	0	33	–	9	13	11	0.2956	18	11	4	0.3905
JXTD	27	1	3	23	0.1856	8	13	6	0.7069	20	6	1	0.4544
JXXW	26	3	5	18	0.1984	15	8	3	0.3196	26	0	0	–
LZSK	31	0	0	31	–	14	14	3	0.4602	31	0	0	–
NPPM	28	0	0	28	–	8	15	5	0.7012	17	10	1	1.0000
PXSS	32	0	1	31	–	15	12	5	0.4321	18	13	1	0.6416

*HWE* Hardy Weinberg equilibrium

The values are p value by the probability test. n = sample size. The abbreviations for the sampling location are given in Fig. 3

The three possible genotypes for site 327 were detected in all the seven populations, and all genotypes agreed with Hardy–Weinberg equilibrium (Table 2). The frequency of the mutant 327I ranged from 26.9 to 53.2% (Fig. 3). The mutant allele (345S) was detected in five of the seven populations, but absent in DXEC and CZSK. Overall, the frequency of 345S was low (0–28.8%), with the highest frequency being observed in FCGDX (Fig. 3).

### Frequency and distribution of triple-locus genotype combinations

When considering the three resistance-related sites together, 11 types of triple-locus genotypes (C1 to C11) were observed (Table 3). The number of triple-locus genotype combinations observed in each sampling site ranged from 3 (CZSK) to 9 (JXTD). Three combinations (C1, C4, C8) were distributed in all the seven populations, while C6 and C7 were uniquely found in PXSS and FCGDX, respectively. The triple-locus wild genotype (C11) was distributed in JXTD and JXXW. The alleles carrying 327I were found only in individuals that harbor 296S, and 345S existed in individuals that were homozygous for 296S. Notably, the triple-locus mutant allele was detected (C6 and C7), although at low frequencies.

**Table 3:** Distribution and frequency of triple-locus genotype combinations in seven *An. sinensis* populations along the China–Vietnam border

Combination	Residue sites			Frequency						
	296	327	345	DXEC	FCGDX	JXTD	JXXW	LZSK	NPPM	PXSS
C1	SS	VV	TT	0.194	0.030	0.074	0.308	0.452	0.107	0.188
C2	SS	VV	TS		0.121	0.074			0.143	0.250
C3	SS	VV	SS		0.121	0.036			0.036	
C4	SS	VI	TT	0.516	0.212	0.300	0.269	0.452	0.321	0.188
C5	SS	VI	TS	0.032	0.182	0.148			0.214	0.156
C6	SS	VI	SS							0.031
C7	SS	II	TS		0.031					
C8	SS	II	TT	0.258	0.303	0.222	0.115	0.096	0.179	0.156
C9	AS	VV	TT			0.074	0.155			0.031
C10	AS	VI	TT			0.036	0.038			
C11	AA	VV	TT			0.036	0.115			

### Evolutionary origin of 296S and 327I

**Fig. 4:**
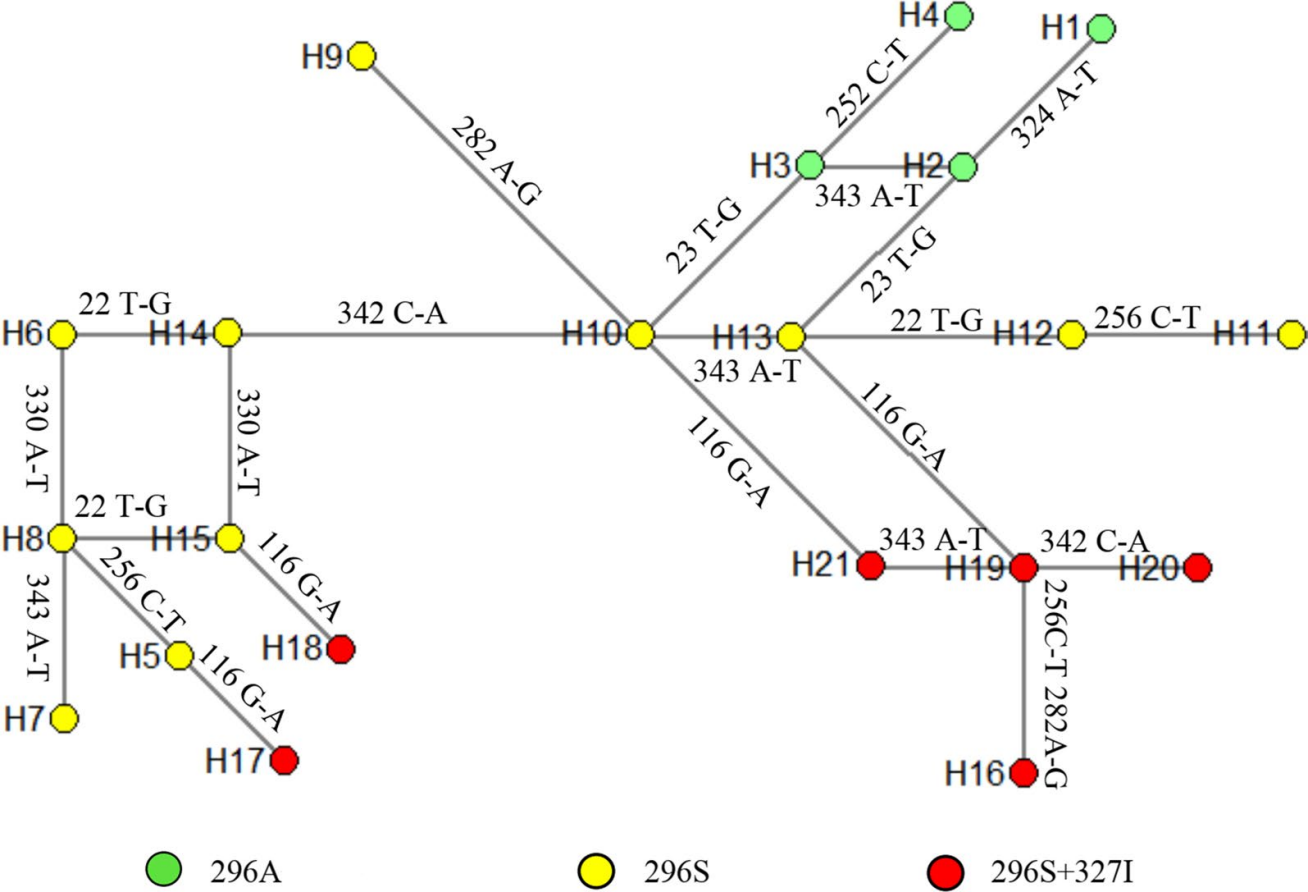
The network of RDL haplotypes identified in *An. sinensis* populations along the China–Vietnam border

To track the possible origin of mutant 296S and 327I, the DNA sequences of 360 bp-in-length (Fig. 2) were used for haplotype identification and network analysis. A total of 21 haplotypes (H1 to H21) were identified (Table 4). Among them, 4 (H1 to H4) were wild haplotypes without 296S and 327I mutations, 11 (H5–H15) were haplotypes with only the 296S mutation, and 6 (H16–H21) carried both 296S and 327I mutations. The 327I mutation was found always together with 296S. Network analysis indicated that the 296S allele might have evolved independently from more than one origin (Fig. 4). The single mutation haplotypes H13 and H10 may be evolved from H2 and H3, respectively, while the five double-mutation haplotypes (H17–H21) could be derived from some 296S haplotypes via only one mutational step (Fig. 4).

**Table 4:** *RDL* haplotypes in seven *An. sinensis* populations along the China–Vietnam border

Haplotype name	GenBank ID	Polymorphic sites	Residue 296	Residue 327
H1	MK686613	TGGTCGTTCA	A	V
H2	MK686614	TGGTCGATCA	A	V
H3	MK686615	TGGTCGATCT	A	V
H4	MK686616	TGGCCGATCT	A	V
H5	MK686617	GTGTTGAAAT	S	V
H6	MK686618	GTGTCGATAT	S	V
H7	MK686619	GTGTCGAAAA	S	V
H8	MK686620	GTGTCGAAAT	S	V
H9	MK686621	TTGTCAATCT	S	V
H10	MK686622	TTGTCGATCT	S	V
H11	MK686623	GTGTTGATCA	S	V
H12	MK686624	GTGTCGATCA	S	V
H13	MK686625	TTGTCGATCA	S	V
H14	MK686626	TTGTCGATAT	S	V
H15	MK686627	TTGTCGAAAT	S	V
H16	MK686628	TTATTAATCA	S	I
H17	MK686629	GTATTGAAAT	S	I
H18	MK686630	TTATCGAAAT	S	I
H19	MK686631	TTATCGATCA	S	I
H20	MK686632	TTATCGATAA	S	I
H21	MK686633	TTATCGATCT	S	I

The polymorphic sites in intron 7 are underlined

## Discussion

Point mutations (A to S/G/N) at the site equivalent to 301 in *Drosophila* (site 296 of AsRDL) in the second transmembrane domain of RDL have been documented in diverse insecticide-resistant insects [10–12, 17–19]. In this study, the resistant mutation 296S was detected and its frequency was very high (79–100%) in the seven examined populations, suggesting a strong risk of resistance to GABA targeting insecticides in these mosquito populations. The prevalence of mutation at 296 was also detected in *An. sinensis* collected in other locations of Guangxi [13], and in *Anopheles funestus* in Africa [12]. Given that the use of cyclodienes has been banned, the high level of 296S in *An. sinensis* in Guangxi may be a consequence of the increasing use of insecticides targeting the GABA receptor (e.g. ethiprole, fipronil) in the agriculture [20], or selection pressures by organochlorine pesticides from unknown origins. Another explanation may be that A296S substitution has no fitness cost under current natural conditions, or the cost mediated by A296S is alleviated by unknown fitness modifiers.

Two mutations (V327I and T345S) previously detected in another nine *An. sinensis* populations from Guangxi [13] were also found in the current study. Interestingly, either 327I or 345S was present together with 296S. The 327I mutation was also previously identified in dieldrin-resistant *An. funestus* [12]. Similarly, a conserved mutation at the equivalent site of RDL was also reported in *Anopheles gambiae* (T345M) [8] and *Drosophila* (T350M/S) [21, 22]. Although the two conserved mutations have been suggested to play a role in insecticide resistance when they occurs together with an amino acid substitution at the site 296 [12, 22], their impact on the pharmacology of the GABA receptor and contribution to insecticide resistance remains unclear. This would be an interesting question worthy of study.

Network analysis does not support single origin of the resistance-conferring 296S mutation and the related 327I mutation in *AsRDL*. The two wild A296 haplotypes (H1 and H4) could be considered as two possible ancestors of resistant 296S haplotypes identified in this study (Fig. 4). Multiple origins of 345S were also inferred in the previous study [13].

## Conclusion

A PCR-RFLP diagnostic assay was developed to genotype the T345S mutation in the *AsRDL* gene. Three previously reported mutations (A296S, V327I and T345S) were detected in the seven *An. sinensis* populations in Guangxi along the China–Vietnam border. The prevalence of the resistance-related A296S mutation within *An. sinensis* populations along the China–Vietnam border indicates a risk of resistance to insecticides targeting the GABA receptor, such as dieldrin and fipronil. The resistance A296S allele may have multiple evolutionary origins, and the double mutations (A296S + V327I) may have evolved from alleles carrying the A296S mutation by scaffolding the additional mutation V327I.

## Data Availability

All the datasets are presented in the main paper. The newly generated sequences were submitted to the GenBank database under the accession numbers MK686613–MK686633.

## Abbreviations

GABA: gamma-aminobutyric acid
Guangxi: Guangxi Zhuang Autonomous Region, China
HWE: Hardy–Weinberg equilibrium
PCR: polymerase chain reaction
RFLP: restriction fragment length polymorphism
RDL: resistant to dieldrin

## References

[1] WHO (2018). World malaria report 2018.

[2] Li JH, Li J, Qin YX, Guo CK, Huang YM, Lin Z (2014). Appraisal of the effect and measures on control malaria for 60 years in Guangxi. J Trop Med..

[3] Li JH, Wei SJ, Yang YC, Li J (2015). Analysis of the prevalence of imported malaria in Guangxi from 2012 to 2013. J Pathog Biol..

[4] Goldlust SM, Thuan PD, Giang DDH, Thang ND, Thwaites GE, Farrar J (2018). The decline of malaria in Vietnam, 1991–2014. Malar J..

[5] WHO (2016). Global technical strategy for malaria 2016–2030.

[6] Yang C, Feng X, Liu N, Li M, Qiu X (2019). Target-site mutations (AChE-G119S and *kdr*) in Guangxi *Anopheles sinensis* populations along the China–Vietnam border. Parasit Vectors..

[7] Casida JE, Durkin KA (2015). Novel GABA receptor pesticide targets. Pestic Biochem Physiol.

[8] Taylor-Wells J, Brooke BD, Bermudez I, Jones AK (2015). The neonicotinoid imidacloprid, and the pyrethroid deltamethrin, are antagonists of the insect Rdl GABA receptor. J Neurochem.

[9] Buckingham S, Ihara M, Sattelle DB, Matsuda K (2017). Mechanisms of action, resistance and toxicity of insecticides targeting GABA receptors. Curr Med Chem.

[10] Ffrench-Constant RH, Rocheleau TA, Steichen JC, Chalmers AE (1993). A point mutation in a *Drosophila* GABA receptor confers insecticide resistance. Nature.

[11] Du W, Awolola TS, Howell P, Koekemoer LL, Brooke BD, Benedict MQ (2005). Independent mutations in the *Rdl* locus confer dieldrin resistance to *Anopheles gambiae* and *Anopheles arabiensis*. Insect Mol Biol.

[12] Wondji CS, Dabire RK, Tukur Z, Irving H, Djouaka R, Morgan JC (2011). Identification and distribution of a GABA receptor mutation conferring dieldrin resistance in the malaria vector *Anopheles funestus* in Africa. Insect Biochem Mol Biol.

[13] Yang C, Huang Z, Li M, Feng X, Qiu X (2017). RDL mutations predict multiple insecticide resistance in *Anopheles sinensis* in Guangxi, China. Malar J..

[14] Edgar RC (2004). MUSCLE: multiple sequence alignment with high accuracy and high throughput. Nucleic Acids Res.

[15] Rousset F (2008). GENEPOP ‘007: a complete re-implementation of the GENEPOP software for Windows and Linux. Mol Ecol Resour..

[16] Bandelt H, Forster P, Rohl A (1999). Median-joining networks for inferring intra specific phylogenies. Mol Biol Evol.

[17] Nakao T, Kawase A, Kinoshita A, Abe R, Hama M, Kawahara N (2011). The A2′N mutation of the RDL γ-aminobutyric acid receptor conferring fipronil resistance in *Laodelphax striatellus* (Hemiptera: Delphacidae). J Econ Entomol.

[18] Nakao T (2017). Mechanisms of resistance to insecticides targeting RDL GABA receptors in planthoppers. Neurotoxicology..

[19] Wei Q, Wu SF, Gao CF (2017). Molecular characterization and expression pattern of three GABA receptor-like subunits in the small brown planthopper *Laodelphax striatellus* (Hemiptera: Delphacidae). Pestic Biochem Physiol.

[20] Wang Y, Wang M (2007). Factors affecting the outbreak and management tactics of brown planthopper in China recent years. Pestic Sci Adm..

[21] Le Goff G, Hamon A, Berge J-B, Amichot M (2005). Resistance to fipronil in *Drosophila simulans*: influence of two point mutations in the RDL GABA receptor subunit. J Neurochem.

[22] Remnant EJ, Morton CJ, Daborn PJ, Lumb C, Yang YT, Ng HL (2014). The role of Rdl in resistance to phenylpyrazoles in *Drosophila melanogaster*. Insect Biochem Mol Biol.

